# Angiotensin Converting Enzyme Inhibitor Has a Protective Effect on Decompression Sickness in Rats

**DOI:** 10.3389/fphys.2018.00064

**Published:** 2018-03-01

**Authors:** Aleksandra Mazur, Anthony Guernec, Jacky Lautridou, Julie Dupas, Emmanuel Dugrenot, Marc Belhomme, Michael Theron, François Guerrero

**Affiliations:** EA4324 ORPHY, Institut Brestois Santé Agro Matière, Université de Bretagne Occidentale, Brest, France

**Keywords:** renin-angiotensin system, calcium channel blocker, decompression illness, vasomotion, animal model, angiotensin converting enzyme inhibitor, angiotensin receptor antagonist

## Abstract

**Introduction:** Commercial divers, high altitude pilots, and astronauts are exposed to some inherent risk of decompression sickness (DCS), though the mechanisms that trigger are still unclear. It has been previously showed that diving may induce increased levels of serum angiotensin converting enzyme. The renin angiotensin aldosterone system (RAAS) is one of the most important regulators of blood pressure and fluid volume. The purpose of the present study was to control the influence of angiotensin II on the appearance of DCS.

**Methods:** Sprague Dawley rats have been pre-treated with inhibitor of angiotensin II receptor type 1 (losartan; 10 mg/kg), angiotensin-converting enzyme (ACE) inhibitor (enalapril; 10 mg/kg), and calcium-entry blocker (nifedipine; 20 mg/kg). The experimental groups were treated for 4 weeks before exposure to hyperbaric pressure while controls were not treated. Seventy-five rats were subjected to a simulated dive at 1000 kPa absolute pressure for 45 min before starting decompression. Clinical assessment took place over a period of 60 min after surfacing. Blood samples were collected for measurements of TBARS, interleukin 6 (IL-6), angiotensin II (ANG II) and ACE.

**Results:** The diving protocol induced 60% DCS in non-treated animals. This ratio was significantly decreased after treatment with enalapril, but not other vasoactive drugs. Enalapril did not change ANG II or ACE concentration, while losartant decreased post dive level of ACE but not ANG II. None of the treatment modified the effect of diving on TBARS and IL-6 values.

**Conclusion:** Results suggests that the rennin angiotensin system is involved in a process of triggering DCS but this has to be further investigated. However, a vasorelaxation mediated process, which potentially could increase the load of inert gas during hyperbaric exposure, and antioxidant properties were excluded by our results.

## Introduction

Decompression sickness (DCS) is the most serious danger for Self-Contained Underwater Breathing Apparatus (SCUBA) divers. It is a systemic pathology displaying a wide range of symptoms including minor ones such as skin rashes up to more serious clinical outcomes like neurological damage, cardiac collapse and death (Vann et al., 2011). Spinal cord DCS, which is the more severe form of DCS, represents 40–45% of total DCS cases and manifests in a broad array of symptoms that can result in severe morbidity, life-long disabilities, and even death. Among these most serious cases, 20–30% of divers will suffer definitive sequelae. DCS is strongly connected with vascular bubble formation resulting from supersaturation during inadequate decompression (Eftedal et al., 2007). However, it has been stated that only 13% of its appearance can be explained by these venous gas emboli (VGE) alone (Ljubkovic et al., 2010), and even high amounts of circulating bubbles do not necessarily lead to DCS (Bakovic et al., 2008). This shows that although necessary the presence of bubbles is not sufficient to evoke DCS.

One possible, and repeatedly hypothesized, mechanism could rely on the well-documented diving-induced impairment of vascular function (Brubakk et al., 2005; Lambrechts et al., 2013a; Mazur et al., 2016). In line with this hypothesis, administration of nitric oxide (NO) donors decreases both the amount of circulating bubbles (Dujić et al., 2006; Møllerløkken et al., 2006) and the probability of DCS (Wisløff et al., 2004), while blockade of NO production increases the probability of DCS (Wisloff et al., 2003; Mazur et al., 2014). In addition, pretreatment with the PDE5 inhibitor sildenafil led to increased occurrence of DCS (Blatteau et al., 2013) which suggests that the level of circulating NO rather than its vasodilating action could be involved in the development of DCS. However, circulating levels of NO assessed after diving failed to show any modification. Indeed, blood nitrate concentration was not different after a single open water sea dive with air or nitrox (Marinovic et al., 2012; Lambrechts et al., 2013b; Theunissen et al., 2013) compared with before the dive.

The renin angiotensin aldosterone system (RAAS) is one of the most important regulators of blood pressure, fluid volume, and sodium and potassium balance. Angiotensin II (Ang II) is produced by the conversion of Angiotensin I (ANG I) by the angiotensin converting enzyme (ACE), an enzyme which is abundant in the lungs, bound to endothelial cells and is also found in many other organs including the kidney. Increased levels of serum ACE have been reported after decompression and at even higher levels following DCS (Thorsen et al., 2006). Although not the only peptide formed by RAAS, Ang II is the major active metabolite and exerts its effects via AT1 and AT2 receptors. Stimulation of AT1 receptors by Ang II is responsible for Ang II evoked contraction of smooth muscle cells, enhancement of sympathetic outflow and release of aldosterone by the adrenal gland. AT1 receptor may also increase oxidative stress and indirectly reduce bioavailabilty of the greatly vasoactive NO (Newsholme et al., 2010), promote formation of endothelial microparticules (Yang et al., 2014) and inflammation-related processes (Pacurari et al., 2014). AT2 receptors stimulate the generation of procoagulant microparticles (Cordazzo et al., 2013) and promote thrombosis in the microcirculation (Senchenkova et al., 2010). All of these have been proven to trigger diving-related physiological changes. Indeed, altered vascular permeability (Gempp et al., 2013), platelet aggregation (Pontier et al., 2012), release of microparticles and inflammation (Thom et al., 2015), as well as oxidative stress (Mazur et al., 2016) have been evidenced post-dive. Recently, we found that plasmatic concentration of AngII was decreased after the dive in asymptomatic rats but not animals which suffered DCS (Mazur et al., 2016). This led us to hypothesize that maintaining the concentration of circulating Ang II could be part of the mechanisms leading to DCS.

That is why in this study we decided to control its influence on appearance of DCS events after a simulative dive *in vivo* in male Sprague Dawley rats. ANG II was blocked on two different levels: production by long-term angiotensin converting enzyme inhibitor with enalapril and its direct action through angiotensin II receptor type 1 by long-term inhibition of AT1 with losartan. Treated rats were compared with the control not-treated group. To further assess whether vasomotion-related mechanisms are involved we included the effect of long-term calcium-entry blockade, assessed by nifedipine treatment, which would show non-ANG II, non-NO regulation of vasomotion and its importance in decompression sickness appearance after a simulative *in vivo* dive.

## Methods

All experiments were conducted in accordance with the Guide for the Care and Use of Laboratory Animals published by the US National Institutes of Health (National Institutes of Health Publication No. 85-23, revised 1996), and national laws from the French Ministry of Agriculture. They were formally approved by the Université de Bretagne Occidentale animal research ethics committee.

### Animals

Eighty-three male Sprague Dawley rats were obtained from Janvier SAS (France) 5 weeks before exposure to pressure. Before treatment the rats were allowed to become accustomed to the facility for a week. They were housed in an environmentally controlled room (temperature 21 ± 1°C, relative humidity 27 ± 16%, 12–12 h light-dark cycle) and fed standard rat chow.

Animals were randomly divided into six groups. Four groups, based on pharmaceutical intervention, were compared post-dive with the fifth, untreated diving control group for susceptibility to DCS. To control for the influence of either diving itself or treatment before diving on plasmatic markers, an additional untreated non-diving control group (*n* = 8) was included.

### Drugs

The treatment lasted 4 weeks and drugs were administrated orally in drinking water. Enalapril (Enalapril EG 20 mg) as an ACE inhibitor (*n* = 15) and losartan (TEVA) as an AT1 receptor antagonist (*n* = 15) were dissolved directly in tap water and given at the dose of 10 mg/kg/day, a dose that has been demonstrated to cause maximal inhibition of ACE activities or blockade of AT1 receptors (Matsuyama and Kitani, 1996). Nifedipine (Sigma Aldrich, Paris, France) as a Ca^2+^ channel blocker (*n* = 15) was administrated at the dose of 20 mg/kg/day (Cao et al., 2010). Due to its low in-water solubility this drug was first dissolved in absolute ethanol (final alcohol concentration 1.2%vol.vol^−1^). To discount any confounding influence of alcohol, an additional alcohol control group (*n* = 15) was created, where 1.2% vol.vol^−1^ ethanol was added to drinking water given to rats. Fresh solutions of all drugs were prepared daily and consumption was monitored. Once per week body weight and systolic blood pressure were measured. After 4 weeks of treatment the rats were placed in a 130-liter hyperbaric chamber (Comex, Marseille, France) and performed a simulated dive.

### Blood pressure measurement

Resting caudal artery Systolic Blood Pressure (SBP) was obtained indirectly by the tail photoplethysmographic technique (IITC INC/Life Science Instruments, Woodland Hills, USA). All rats underwent 1 week of habituation to the procedure prior to the experiment. Measurements were made in front of a radiator at 29–30°C to vasodilate the tail artery. In each group baseline SBP was measured once during the treatment period and then again on the day before each compression protocol. Presented values of SBP are the mean of three separate readings obtained during the last measurement. Mean SBP values after the treatment, but before compression, were compared with those obtained in control animals.

### Simulated dive protocol

Because both age and weight are known to influence the probability of DCS (Buzzacott et al., 2016), animals used in these experiments were all the same age (12 weeks old) and similar weights (450 ± 50 g) on the day of the experiment. The simulated dive protocol has been previously described (Mazur et al., 2016). Briefly, rats from each group were placed in a dry hyperbaric chamber. Compression and decompression were at a rate of 100 kPa.min^−1^. Diving rats were compressed with air up to 1000 kPa absolute pressure and remained under this pressure for 45 min. Three decompression stops were performed during the ascent: 5 min at 200 kPa, 5 min at 160 kPa, and 10 min at 130 kPa. Total duration of the hyperbaric exposure was 83 min. All dive depths were monitored using a modified personal dive computer (Puk, Mares, Rapallo, Italy). This protocol has been used in our previous experiments and reliably induces about 63% of DCS. A non-diving control group of rats was similarly confined, (but not exposed to elevated ambient pressure), and observed for 1 h before physiological investigation. Following the observation period the rats were anesthetized by intramuscular injection of ketamine (80 mg/kg) and xylazin (15 mg/kg). After blood and organs had been collected the rats were euthanized with pentobarbital.

An additional 48 control rats from a previous experiment were added to the 15 control rats in this study, thus raising the control to treatment ratio to 4:1. These rats underwent the same diving protocol, were handled the same way, had similar weight and were the same age on the day of the simulative dive.

### Classification of DCS

Animals were scored as having DCS only when displaying one or more symptoms of: respiratory distress, difficulty walking, paralysis, or convulsions (Arieli et al., 2009). The classification of DCS and analytical method were decided *a priori* to the experiment. To minimize the potential for animal suffering a pain/distress scale was approved by the Animal Research Ethics Committee and no rats displayed signs of distress at or above the level where early euthanasia was required. In nearly all non-fatal cases of DCS the rats appeared to recover fully by the end of the observation period, to the point where they were indistinguishable from rats without DCS. Other studies using an ED-50 rat model have classified their outcome variables as DCS vs. No-DCS, whereby the marginal cases were combined with the dead, or the classification was Dead vs. Not-Dead, where the marginal cases were combined with the asymptomatic. In this study the ternary classification of “No DCS” (no symptoms), mild “DCS” (with at least one symptoms excluding death within the observation period) or severe DCS (“Death” within 1 h) was retained to maximize statistical power, as described by Buzzacott et al. (2014).

### Blood sampling and plasmatic markers

Blood samples were collected immediately following anesthesia or death in a 2 mL eppendorf tube with 30 μl 7.5% EDTA as an anticoagulant, centrifuged at 1000 g and 4°C for 15 min, aliquoted and stored at −80°C until the assay. To explore which regulation loop was involved in DCS appearance we measured different biomarkers in plasma from the non-diving, diving with “No DCS” and mild “DCS” groups. Thiobarbituric Acid Reactive Substances (TBARS) was chosen as an indicator of oxidative stress and Interleukin 6 (IL-6), a pleiotropic cytokine associated with the inflammation process. Additionally, levels of angiotensin II (ANG II) as a peptide hormone that causes vasoconstriction and ACE an enzyme responsible for conversion of angiotensin I (ANG I) to its active form ANG II was measured to track the influence of the renin-angiotensin system on the appearance of DCS. Plasmatic concentrations were determined using commercially available ELISA assay kits; TBARS Assay Kit (Cayman Chemical Company, Ann Arbor, MI), ACE Assay Kit (EIAab, Wuhan, China) and Il-6 Rat ELISA Kit provided by ABCAM, (Paris, France). All ELISA samples were run in duplicate in accordance with the manufacturer's instructions.

### Statistical analysis

Data were analyzed using SAS ver. 9.3 (SAS, Cary, North Carolina). An ordinal logistic regression model was constructed as shown in Equation 1, using a cumulative logit function appropriate for ordinal, polychotomous dependent variables.

(1) $$\begin{array}{r} DCS=\beta_{0}\;+\;\beta_{1}TREATMENT\;+\;\beta_{2}WEIGHT \end{array}$$

Where DCS was 0 = asymptomatic, 1 = alive for 1 h but with signs of DCS, 2 = dead within 1 h. WEIGHT was the weight of each rat on the day of compression in grams, TREATMENT indicates whether each rat was treated with enalapril, losartan, or not treated (control). A similar but separate model was created for nifedipine which was compared with the alcohol group and also to the non-treatment control group. Data from kit measurements were analyzed using Statistica software (ver. 10, StatSoft France, 2011). Differences between the concentrations of plasmatic ACE, ANG II, TBARS, and IL-6 after the diving simulation were first analyzed for normality. If data were parametric we proceeded with one way ANOVA. Upon identifying significant differences in the ANOVA, a Dunnett *post-hoc* test was used to interrogate relevant parameters. For non-parametrically distributed results we ran ANOVA with a Kruskal–Wallis test. Data were considered significant at *p* < 0.05 and reported as means ± SE for indicated samples.

## Results

### Blood pressure measurements

Systolic Blood pressure decreased in all rats that received antihypertensive drugs. It was significantly lower after the administration of enalapril (98 ± 2.3 mmHg) when compared with the control group (120 ± 2.2 mmHg, *p* < 0.01). Losartan (112 ± 2.9 mmHg) and nifedipine (112 ± 2.2 mmHg) showed a tendency to decrease SBP and the alcohol treatment did not change the blood pressure (117 ± 2.7 mmHg).

### DCS outcome

**Figure 1:**
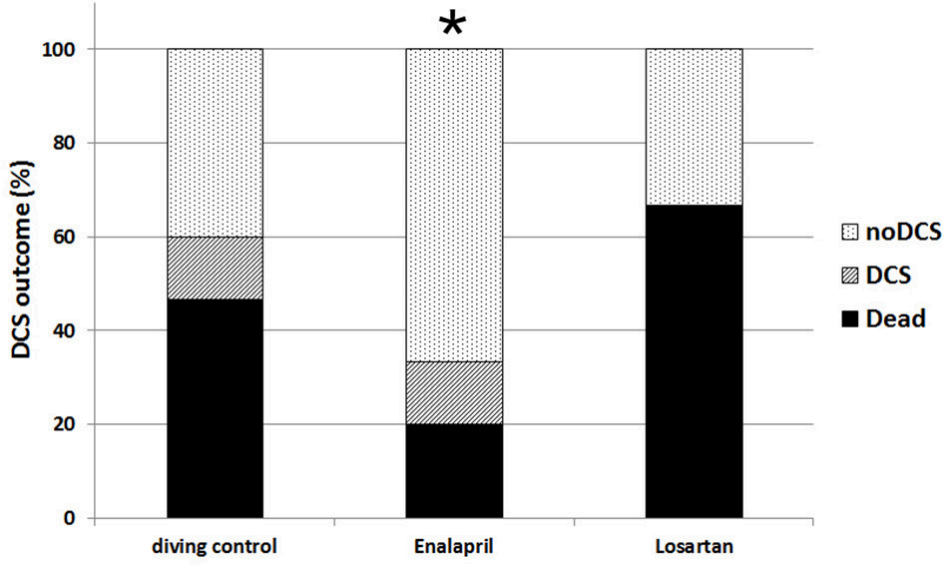
Appearance of DCS among control rats and rats treated with enalapril and losartan. Appearance of DCS among the control rats and rats treated with enalapril and losartan expressed as a percentage of the total number of the rats in each group. Histogram in black represents the rats suffering severe DCS, stripes are rats with mild DCS and dots are the asymptomatic group ^*^p < 0.05.

**Figure 2:**
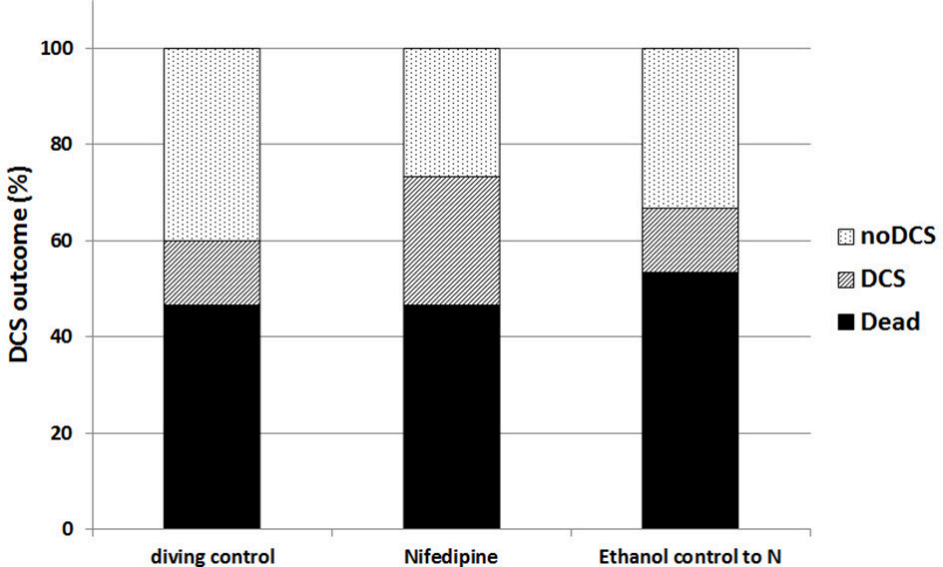
Appearance of DCS among control rats, additional control alcohol group and rats treated with nifedipine. Appearance of DCS among the control rats, additional control alcohol group and rats treated with nifedipine expressed as a percentage of the total number of the rats in each group. Histogram in black represents the rats suffering severe DCS, stripes are rats with mild DCS and dots are the asymptomatic group. There was no statiscally significant difference between groups.

The diving protocol induced 60% DCS in the diving control group (Figure 1). Death appeared in 77% of the affected rats before the end of the observation period (usually during the last minutes of ascent or within 30 min after hyperbaric exposure). The remaining 33% of the affected rats all survived the observation period. The additional control group from a previous experiment was not significantly different in DCS outcome to the 15 control rats in this experiment, therefore these groups were merged. Due to its narrow range between animals, body weight had no significant influence upon DCS outcome in this experiment. Pre-treatment with enalapril lowered the ratio of DCS when compared with combined diving controls (*p* = 0.01). Enalapril also appeared to be significantly protective compared to losartan, which did not differ from the combined diving controls. Treatment with nifedipine did not significantly alter the appearance of DCS (Figure 2). The outcome of DCS in the alcohol group was also no different when compared with the combined diving control rats, which indicates that ethanol itself had no influence on the appearance of DCS (Buzzacott et al., 2015).

### ACE, ANG II, TBARS and IL-6 assays

**Figure 3:**
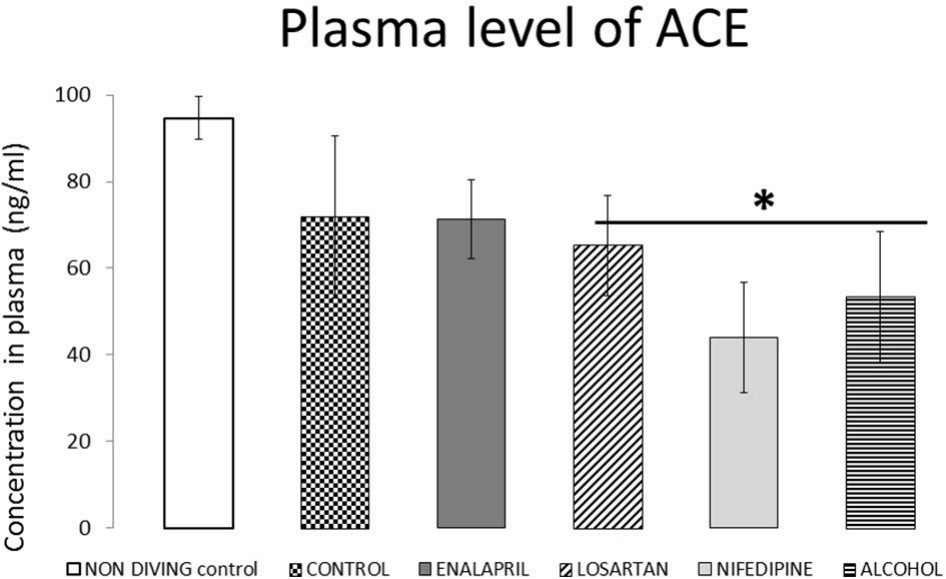
ACE plasma levels for all diving groups with treatment compared with diving control and non-diving rats. ACE plasma levels expressed as ng/ml (mean ± SEM), for all the diving groups with treatment compared with diving control and non-diving rats. ^*^p < 0.05.

Plasma ACE values are presented in Figure 3. Levels of ACE were not significantly different in control diving rats from the non-diving control group. We observed significantly lower post-dive concentrations in animals treated with losartan (*p* = 0.02), nifedipine (*p* < 0.10), or alcohol (*p* < 0.10) when compared with non-diving controls. Conversely, treatment with enalapril did not change the post-dive level of ACE when compared with either non-diving or diving controls.

**Figure 4:**
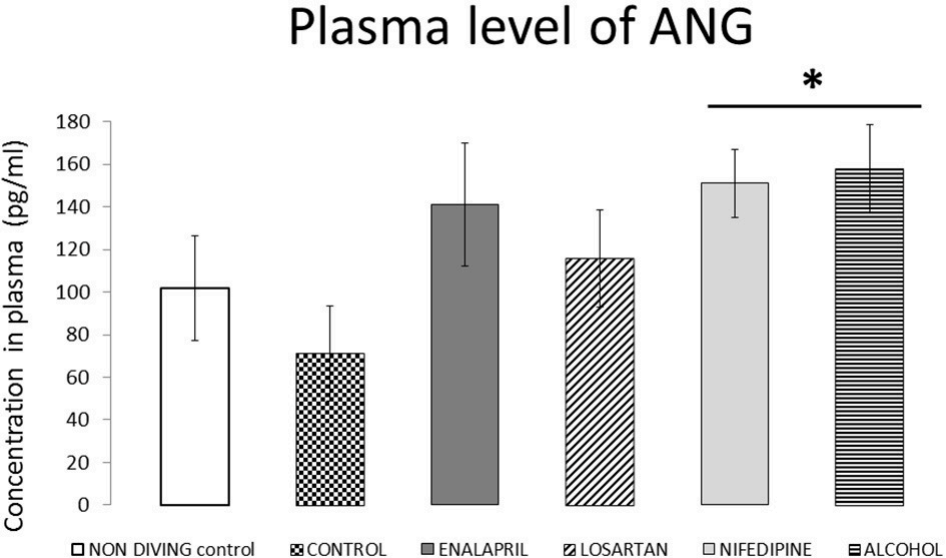
ANG plasma levels for all diving groups with treatment compared with diving control and non-diving rats. ANG plasma levels expressed as pg/ml (mean ± SEM), for all the diving groups with treatment compared with diving control and non-diving rats. ^*^p < 0.05.

Active forms of ANG II values are shown in Figure 4. The diving group did not differ significantly when compared with the non-diving group. Similarly, neither enalapril nor losartan modified post-dive levels of ANG II. Plasmatic concentration of ANG was significantly higher after the dive in rats that received alcohol alone or alcohol and nifedipine than in the diving control group (*p* = 0.01). However, nifedipine did not show a significant effect when compared with the alcohol group, which indicates that alcohol itself significantly elevated levels of ANG.

**Figure 5:**
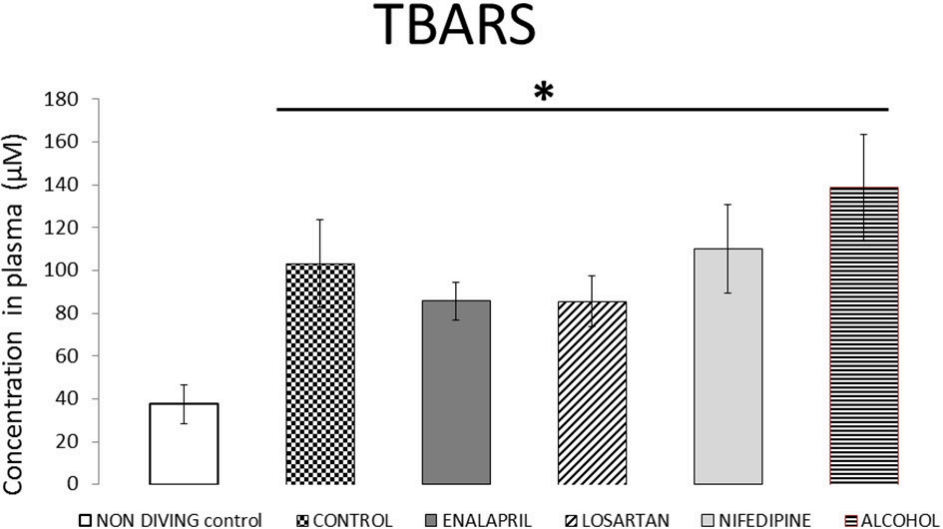
TBARS plasma levels for all diving groups with treatment compared with diving control and non-diving rats. TBARS plasma levels expressed as μM (mean ± SEM), for all the diving groups with treatment compared with diving control and non-diving rats. ^*^p < 0.05.

TBARS values are shown in Figure 5. Their level was significantly elevated among all diving groups when compared with non-diving control rats (*p* < 0.04), But there was no difference between treated groups.

**Figure 6:**
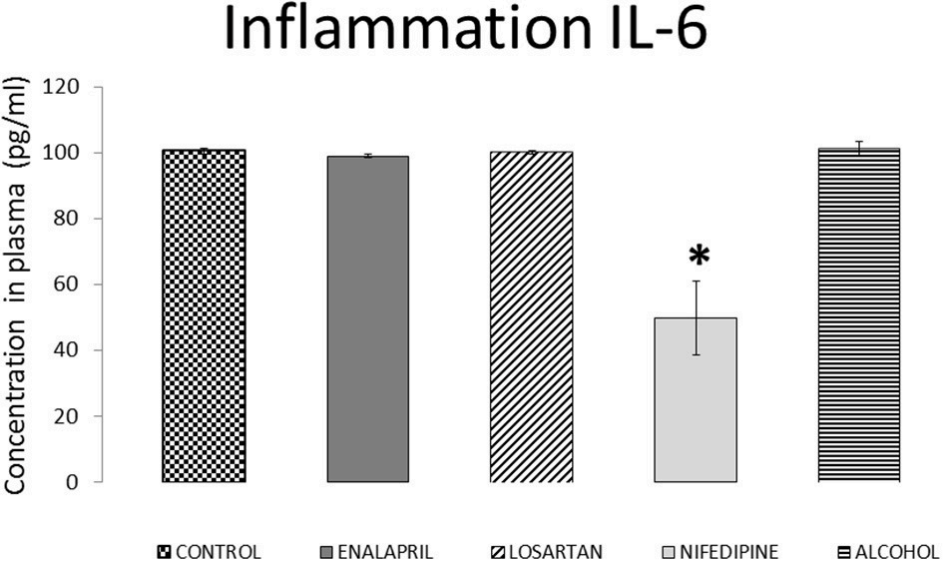
IL-6 plasma levels for all diving groups with treatment compared with diving control. IL-6 plasma levels expressed as pg/ml (mean ± SEM), for all the diving groups with treatment compared with diving control. ^*^p < 0.05.

IL-6 levels (Figure 6) were measured only in the diving groups. IL-6 did not significantly change in enalapril or losartan treated rats when compared with the diving control group. Nifedipine treatment stimulated a significant decrease of IL-6 levels when compared with the alcohol group (*p* = 0.001). Alcohol itself did not differ from basal values when compared with the diving control group (*p* = 0.76).

## Discussion

The main focus of this study was to assess whether the renin angiotensin system has an influence on the appearance of DCS. Additionally, by chronic administration of substances which inhibit either the formation of Ang II or its action on the vessels, or promotes vasomotion independently of the RAAS and NO-cGMP pathways, we aimed to confirm (i): whether the RAAS could be involved in the development of DCS and, if so, (ii): by which mechanisms.

A decrease in systolic blood pressure indicated that all of the drugs were efficiently administrated. However, because enelapril decreased SBP significantly more than either losartan or nifedipine we cannot exclude the hypothesis that lower blood pressure could have been protective against DCS. Also the effect obtained can be a dose related process thus we cannot exclude the scenario that other drugs would influence the outcome of DCS when administrated in a higher doses.

Among the drugs influencing the renin-angiotensin system, the angiotensin converting enzyme inhibitor enalapril appeared to significantly decrease the ratio of accidents while the angiotensin receptor blocker (ARB) losartan did not change the outcome. Such a divergent effect between these two drugs has been recently discussed (Strauss and Hall, 2016). Indeed, the authors claimed that although both drugs lower blood pressure, ACE inhibitors but not ARBs may also produce a pressure independent benefit and reduce the risk of myocardial infarction in people with cardiovascular risk factors. Similarly, we found that only the ACE inhibitor prevented DCS. This difference in susceptibility to DCS may be explained by the differently acting mechanisms of those drugs. Indeed, enalapril block the formation of angiotensin II and, thus, lowers or suppress its concentration while losartan blocks the effect through the AT1 receptor only. Our initial hypothesis leading this study was based on a previous work by our group which showed that the same hyperbaric protocol is associated with decreased plasmatic concentration of Ang II in animals with no signs of DCS while unchanged in the ones which suffer DCS (Mazur et al., 2016). Because ANG II is responsible for a number of diverse actions in the body, many of them connected with diving-physiology such as increased oxidative stress (Newsholme et al., 2010), control of inflammation-related processes (Davis, 2006) and participation in the thrombotic process (López-Farré et al., 2001), we thus hypothesized that keeping the post-dive concentration of ANGII low enough could prevent DCS. To do so, we compared the effect of enalapril with losartan, which do not act on ANGII concentration. That in the present study the plasmatic concentration of Ang II is unchanged in the control group, in which 60% of the animals suffer DCS, confirms these previous data. However, in the present study both enalapril and losartan failed to decrease plasmatic concentration of ANGII. Indeed, according to previous studies, plasmatic ACE level should have increased after enalapril treatment (Costerousse et al., 1998) while plasmatic level of ANG II decreased (Zhang et al., 2012). Though this confirms that the SRAA is probably involved in the triggering of DCS; it also indicates that enalapril acted through other mechanism(s) that the lowering of AngII.

Diving-induced oxidative stress is well-documented. Previous data indicate that the production of reactive oxygen species (ROS) already increases during the stay at depth (Wang et al., 2015a), and that it is further increased by decompression stress in a dose-dependent manner (Mazur et al., 2016). Based on these observations, it was hypothesized that increased oxidative stress could participate to the development of DCS. In our present study, levels of TBARS increased among all diving groups regardless of the treatments, which is consistent with that diving-induced oxidative stress. ANG II may locally produce superoxide-mediated vascular dysfunction (Newsholme et al., 2010) and is known as a pro-oxidative substance (Durand and Lombard, 2013). It has been proven that drugs used in the treatment of cardiovascular risk factors and especially ACE inhibitors have anti-inflammatory properties by acting as antioxidants. They prevent lipoprotein oxidation and nitric oxide quenching (Osiecki, 2004). However, in our study not only TBARS were not decreased by our treatments, but they were also not significantly different in the enalapril treated group than in the others despite significantly different DCS outcomes. This suggests that the protective effect of enalapril treatment is not due to its antioxidant effect. More generally, this result is in line with data from our team showing that the protective effect of anti-agregant is not associated with decreased post-dive oxidative stress (Lambrechts et al., 2015) and that pre-treatment with antioxidant did not prevent the occurrence of DCS in rats (Wang et al., 2015b), thus pointing a lack of relationship between oxidative stress and DCS.

Because inflammation has been repeatedly reported after diving, we also assessed whether enalapril treatment could act through its anti-inflammatory action. Previous studies showed that IL-6 is increased with DCS (Ersson et al., 1998; Blatteau et al., 2012; Bao et al., 2015) but not asymptomatic individuals (Chen et al., 2011; Blatteau et al., 2012). In our study rats treated with enalapril did not have modified levels of this biomarker. Treatment with nifedipine appeared to reduce levels of IL-6. This effect, however, did not change the outcome of DCS among nifedipine treated rats, suggesting that inflammation through IL-6 was not solely responsible for DCS. However there are many other markers which could be involved in establishing the inflammatory effect and they have been not studied here, thus we cannot definitively exclude this hypothesis.

Another property of ACE inhibitors not shared by ANG receptor blockers is their preventive effect on the breakdown of bradykinin. Bradykinin exerts an antithrombotic action through inhibition of both platelet aggregation and circulating PAI-I levels and is a potent stimulator of tissue plasminogen activator. Patients with chronic heart failure that have been treated with ACE inhibitors had reduced fibrinogen and endothelial von Willebrand factor levels when compared with baseline (Gibbs et al., 2001). Decreases in post-dive platelet count have been previously shown (Pontier et al., 2008; Ostrowski et al., 2011). Additionaly pre-treatment with an antiplatelet agent (abciximab) pretreatment has a strong protective effect on decompression risk by significantly improving DCS outcome (Lambrechts et al., 2015). Finally, increased PAI-1 levels (Eftedal et al., 2012) have been previously reported in rats in a context of decompression sickness. Thus, an improvement in prothrombotic state through increased bioavailability of bradykinin following treatment with ACE inhibitors would be consistent with the protective effect of enalapril but not losartan. Furthermore, ACE inhibition increases the release of nitric oxide (NO), which administration has been shown to prevent DCS in animals (Wisløff et al., 2004), through the accumulation of bradykinin (Cheetham et al., 2000). The effects of bradykinin have not been previously studied in either diving or DCS but could be an interesting new path to investigate.

Finally, we also assessed whether the influence of the RAAS on DCS could be related to its influence on vascular tone. We aimed to inhibit contraction through different pathways. Treatment with nifedipine, a specific blocker of L-type voltage dependent Ca^2+^ channels, aimed to inhibit direct vasoconstriction by influx of extracellular Ca^2+^ whereas blockade of the effect of ANG II, a secondary messenger dependent pathway, was obtained by losartan and enalapril. Neither losartan nor nifedipine altered the likelihood of DCS when compared with the combined diving control group, suggesting that the protective effect of enalapril probably not relies on vasodilating properties.

The main finding of this study is that chronic treatment with enalapril significantly protected rats from suffering DCS while neither losartan or nifedipine treatments influenced the outcome of the dive. The mechanism by which enalapril exerted this positive influence is still unclear but we may exclude antioxidant and anti-inflammatory properties as well as increased gas load induced by increased vasorelaxation. We instead hypothesize that this effect could be due to the influence of enalapril on bradykinin. This has to be further investigated before the influence of ANG II upon developing of DCS will be understood.

## Author contributions

AM, AG, JD, MT, and FG: conception and design of research; AM, JL, ED, MB, and FG: performed experiments; AM, AG, and FG: analyzed data; AM, AG, MT, and FG: interpreted results of experiments; AM: prepared figures; AM, MT, and FG: drafted manuscript; AM, AG, JL, MB, MT, and FG: edited and revised manuscript; AM, AG, JL, JD, ED, MB, MT, and FG: approved final version of manuscript.

### Conflict of interest statement

The authors declare that the research was conducted in the absence of any commercial or financial relationships that could be construed as a potential conflict of interest. The reviewer CRO and handling Editor declared their shared affiliation.
